# Biomechanical and functional variation in rat sciatic nerve following cuff electrode implantation

**DOI:** 10.1186/1743-0003-11-73

**Published:** 2014-04-23

**Authors:** Stephen M Restaino, Erkinay Abliz, Kelliann Wachrathit, Victor Krauthamer, Sameer B Shah

**Affiliations:** 1 Fischell Department of Bioengineering, University of Maryland, College Park, MD, USA; 2 Division of Physics, OSEL/CDRH/ United States Food and Drug Administration, Silver Spring, MD, USA; 3 Departments of Orthopaedic Surgery and Bioengineering, University of California, San Diego, La Jolla, CA, USA

**Keywords:** Peripheral nerve, Cuff, Electrode, Electrophysiology, Biomechanics, Electromyography, EMG

## Abstract

**Background:**

Nerve cuff electrodes are commonly and successfully used for stimulating peripheral nerves. On the other hand, they occasionally induce functional and morphological changes following chronic implantation, for reasons not always clear. We hypothesize that restriction of nerve mobility due to cuff implantation may alter nerve conduction.

**Methods:**

We quantified acute changes in nerve-muscle electrophysiology, using electromyography, and nerve kinematics in anesthetized Sprague Dawley rat sciatic nerves during controlled hindlimb joint movement. We compared electrophysiological and biomechanical response in uncuffed nerves and those secured within a cuff electrode using analysis of variance (ANOVA) and regression analysis.

**Results:**

Tethering resulting from cuff implantation resulted in altered nerve strain and a complex biomechanical environment during joint movement. Coincident with biomechanical changes, electromyography revealed significantly increased variability in the response of conduction latency and amplitude in cuffed, but not free, nerves following joint movement.

**Conclusion:**

Our findings emphasize the importance of the mechanical interface between peripheral nerves and their devices on neurophysiological performance. This work has implications for nerve device design, implantation, and prediction of long-term efficacy.

## Introduction

The peripheral nervous system (PNS) provides a conduit for signal transduction throughout the body. An array of biomedical devices that interact with the PNS have been developed for both recording and stimulation. Currently used devices, both clinically and in a research environment, include epineural and intrafascicular designs. Epineural designs include simple cylindrical cuffs, self-sizing spiral cuff electrodes, and elliptically shaped flattening electrodes [1-4]. These electrodes seek to non-invasively (relatively) elicit and record action potentials from whole nerve bundles. In contrast, intrafascicular designs seek to maximize recording and stimulation selectivity of specific fascicular bundles, though at the expense of increased invasiveness. Intrafascicular designs include single shank longitudinal [5-8] and transverse [9], [10] electrodes as well as transverse electrode arrays [11-14].

Among the many device choices, cylindrical cuffs remain a popular, versatile, and successful technology. They have been used to house multiple electrode types, from simple extraneural contacts [1], [2] to multichannel intrafascicular arrays [3]. Clinically, uses for cuff electrodes include stimulation and recording from the optic nerve for retinal prostheses [15-18], vagus nerve for seizure prevention [19-25], the hypoglossal nerve for sleep apnea and phrenic nerves for respiratory modulation [26-29]. Recent work has also led to advances in cuffs that allow high selectivity of fiber bundles in both stimulation and recording of motor unit potentials for use in functional electrical stimulation (FES) devices. These devices seek to provide ambulatory assistance to patients with spinal cord injuries and as signals to control prosthetics for amputees [7], [8], [10], [30-34]. The simplicity and cylindrical shape of the cuff design also lends itself as a guiding conduit in nerve regeneration studies [3], [4].

Despite their advantages and efficacy, some work has shown chronic damage to the nerve and its environment for reasons that have yet to be fully explained. Specifically, several functional and morphological changes have been reported following cuff implantation, including defective nerve conduction, demyelination, axonal degradation, and tissue encapsulation [2], [35-45]. These may be associated with entrapment following local compression or tethering from the pulling effect of lead migration, which have been observed following clinical implantation of cuff electrodes [24], [25], [43], [46]. Under normal physiological conditions, nerves glide and stretch predictably to accommodate the rotation of spanned joints. Especially in joint regions, segments of nerves approach and, paradoxically, exceed strain thresholds at which conduction is reversibly or irreversibly altered. These thresholds are typically estimated to be 8-15%, though there is high variability across and even within species [47-55]. It then follows that implant-associated entrapment or tethering may perturb this dynamic and carefully controlled biomechanical environment.

**Figure 1:**
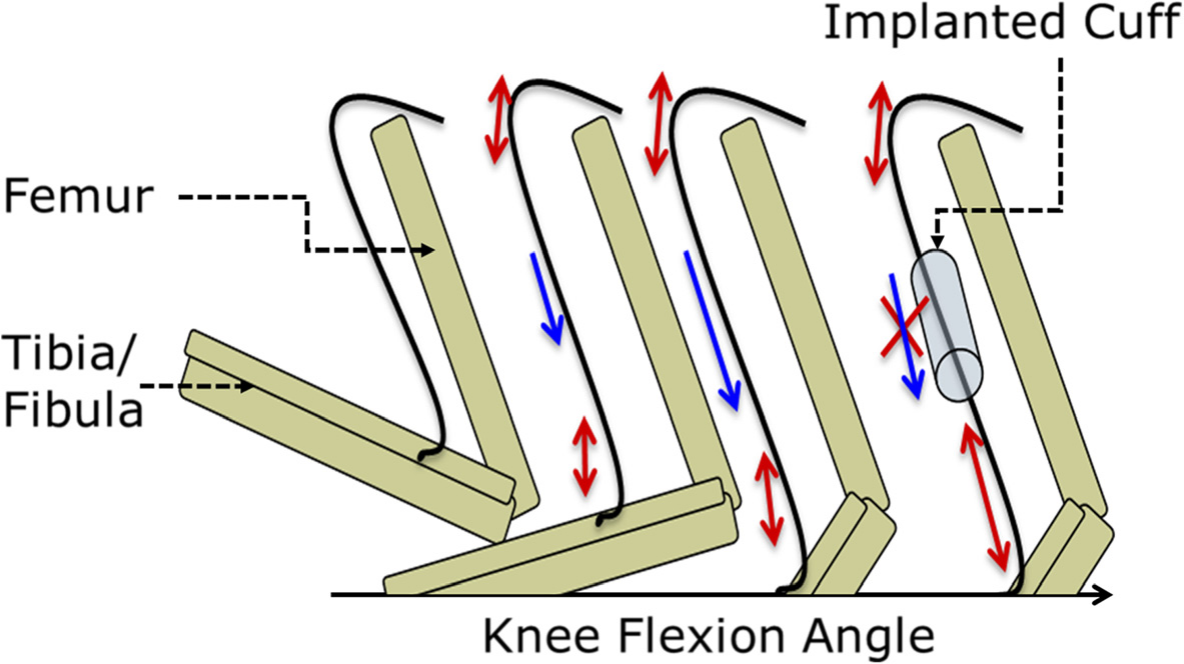
**Schematic of natural and unnatural nerve tensile loading on a sciatic nerve system with and without a nerve cuff implant.** Knee Extension angle increases towards the right of the schematic. Double-headed arrows represent stretching above joint sections. Single-headed arrows represent excursion.

Though longer-term impacts of electrode implantation have been studied, little work has investigated factors that could predict or initiate subsequent dysfunction. We hypothesize that cuff implantation acutely alters the electrophysiological function of a nerve by changing the distribution of tensile loads along the nerve (Figure 1). To test this hypothesis, kinematic analysis and electromyography (EMG) were applied to a rat sciatic nerve model of cuff electrode implantation. Our data revealed increased variability in action potential latency and amplitude, altered nerve alignment, and irregular nerve deformation during joint movement in cuffed rat sciatic nerves compared to uncuffed controls.

## Methods

### Animal use and experimental groups

All procedures were approved by the Institutional Animal Care and Use Committees (IACUC) at the United States Food and Drug Administration and the University of Maryland, College Park. A rat sciatic nerve model was used to test the effects of cuff electrodes on nerve mechanics and function. 14-week old (~400 g), male Sprague-Dawley rats (Taconic, Inc, Rockville, MD) were clustered into two groups, “implant” (n = 19) and “hook” control (n = 6). Implant group nerves were stimulated with a cuff electrode, while the hook group was stimulated with a subminiature hook electrode (Item ID: 501650, Harvard Apparatus). Within these groups, in a “position control” subgroup (n = 5 implant, n = 6 hook), electrophysiological tests were performed in both reversed experimental order (extended to relaxed) and standard order (relaxed to extended).

### Nerve cuff electrode

**Figure 2:**
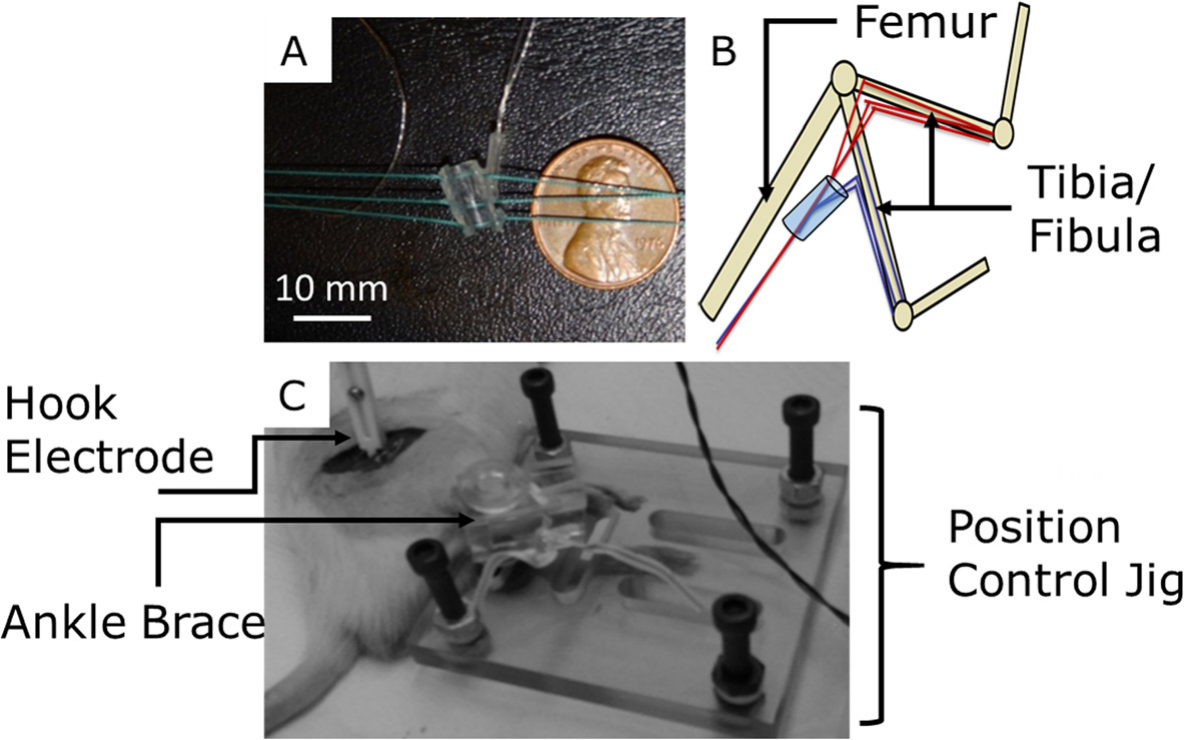
**Image A (Top Left), photograph with dimensions of cuff used for electrophysiology**/**biomechanics testing.** Dimensions: inner diameter, 2 mm; outer diameter, 4 mm; length, 7.5 mm. Image **B** (Top Right), simple schematic of positions used to reach maximum and minimum natural nerve elongation. Image **C** (Bottom), shows mechanical device for maintaining target knee extension angles, the ankle “brace” is for maintaining a constant 90 degree ankle angle.

A commercially available silicon cylindrical nerve cuff (Microprobes, Inc; inner diameter, 2 mm; outer diameter, 4 mm; length, 7.5 mm; Figure 2A) was chosen to represent the typical material and geometry of commercially available implantable cuff electrodes. The chosen device was a bipolar electrode with stainless steel contacts and an inner diameter that could be tightened with integrated suture ties to a diameter matching that of the cuffed nerve, to prevent a static compressive environment. This simple, inexpensive device design allows for consistent implantation and appropriate electrode contact with the nerve.

### Device implantation

#### Pre-surgery

The rats were anesthetized using 2-3% isoflurane, via inhalation, and placed on a heating pad (NECO-819, setting: Medium) to maintain body temperature throughout the procedure. Adequate anesthetic induction was tested throughout surgery via the toe pinch method.

#### Surgery

The initial surgical incision was a proximal-distal cut lateral to the femur path. All subsequent incisions were between muscles or heads of muscles (semitendinosus and quadriceps femoris) to preserve tissue and minimize blood loss. The cuff was implanted approximately 0.5 cm proximal to the trifurcation of the sciatic nerve per methods carefully detailed in the literature, with the rat knee in a neutral configuration [26], [56]. Care was also taken to align the cuff parallel to the long-axis of the nerve during surgery, and the cuff was tightened to create a secure interface with the underlying nerve. Examination of the distal edge of the cuff at 3×-10× magnification confirmed that there was no apparent lateral or anterior nerve bulge indicative of compression. After cycling joints through their range of motion, we confirmed that there was no cuff slippage or translation based on position of distal cuff edge relative to original position on nerve, and that the cuff returned to its original alignment in a neutral configuration. Two scientists (SMR and EA) independently performed implantations, with validation by the other scientist to ensure consistency. All subsequent measurements were made immediately following completion of implantation procedures, with the entire experiment completed within 30 minutes. Following device implantation, all surgical wounds were closed temporarily with a hemostat, allowing the electrode wires to pass through the incision, but better simulating the in vivo constraints of an intact nerve bed during electromyography or joint positioning. After joint positioning, nerves were exposed for digital photography.

### Biomechanics

**Figure 3:**
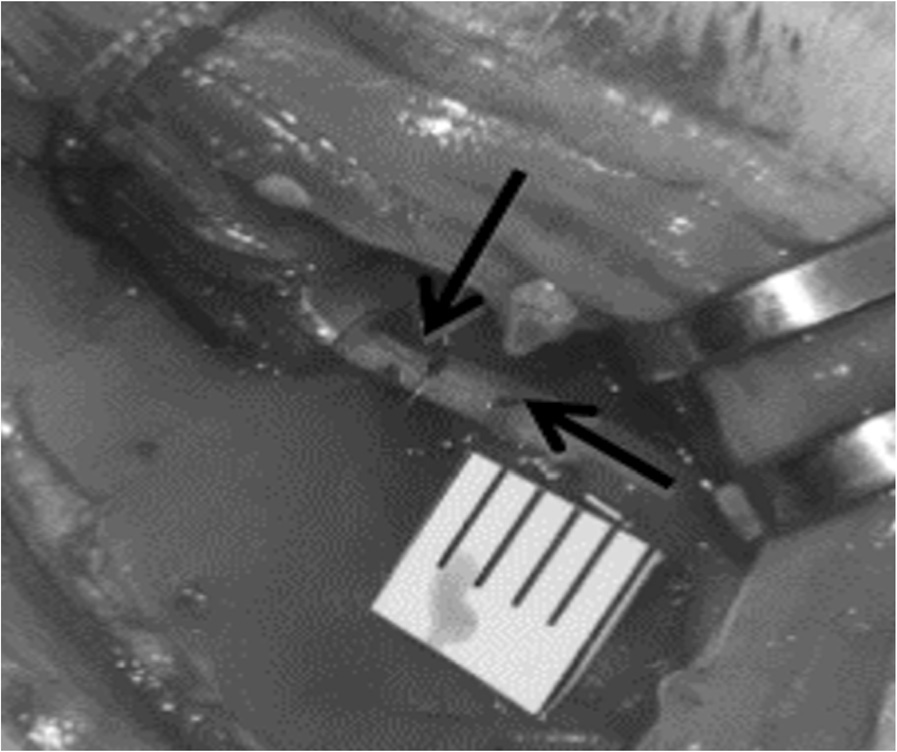
**Photograph taken of nerve marking procedure.** 7-0 monofilament sutures were tied into a square knot through the epineurium. Changes in strain were measured as a distance change between the suture markers. Knot locations are marked with arrows.

Strain measurements were made distal to the nerve cuff. In order to quantify nerve strain, nerves were marked in-vivo with a square knot of 7-0 monofilament suture through the epineurium in both cuffed and control nerves (Figure 3). Suturing did not evoke a muscle twitch, indicating that the suture was confined to the epineurium. Also, suture regions did not appear damaged after experimentation, and it is unlikely that suturing appreciably affected the kinematic response, as the perineurium is believed to bear the majority of physiological loads [48], [57]. Measurements were taken in a relaxed position, full knee flexion, and in a fully extended position, full knee extension. These two positions maximized the change in nerve excursion and strain within a normal physiological range, via manipulation of a single joint (Figure 2B). The hindlimb was secured in a custom-fabricated jig to maintain a relaxed hip flexion angle of 60 degrees, ensure consistency of joint position from animal to animal, and minimize electrode movement (Figure 2B & 2C). Limb positions were held only for the time necessary for images (or EMG recordings, see *Electromyography* section) to be taken. Measurements of knot centroids were made post hoc in MATLAB™ from digital photographs taken during the procedure. Strain was calculated as a percent change in marker separation along the nerve axis from the relaxed to the extended position. Strain measurements were taken as an average of 5 repeated measurements to reduce human error; error between measurements was less than 1 pixel (~1.5%).

### Electromyography

**Figure 4:**
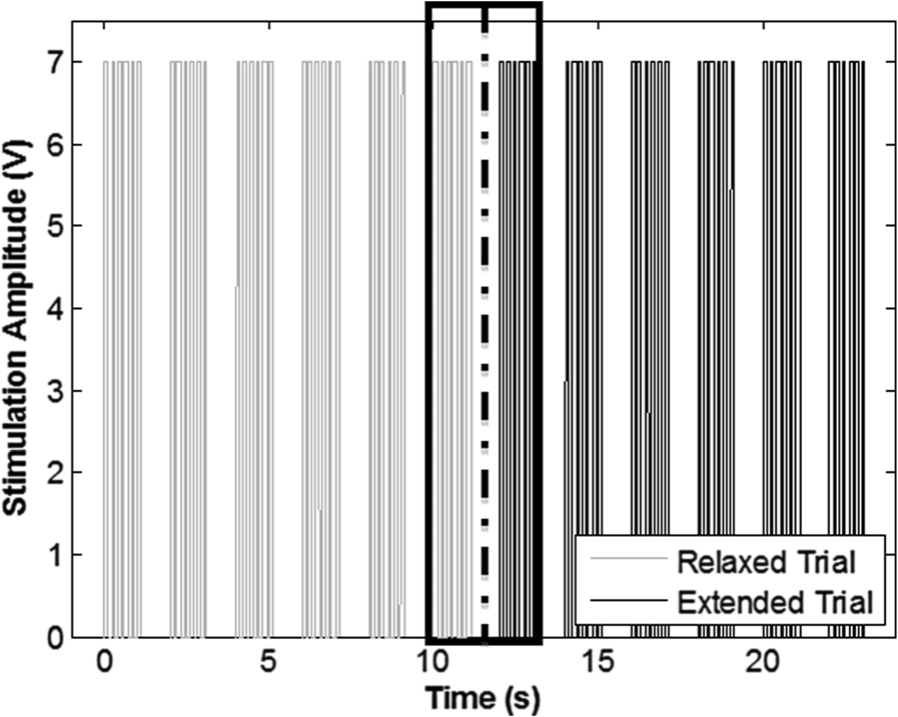
**Plot of stimulation sets used in studying response latency.** Stimulations were set in 5 trains of 6 stimulations for each position. The dotted line represents the point at which the knee was rotated from flexed to extended and vice versa. The boxed trains represent the trains used for calculations. These trains were chosen to eliminate time dependent factors in response amplitude caused by fatigue.

EMG was utilized to record the twitch amplitude and latency following the applied stimuli. EMG was chosen over direct nerve recording to prevent additional surgical procedures that would be necessary to isolate a neural recording site, which may further disrupt the mechanical environment of the nerve. The rat sciatic nerve was stimulated by the implanted nerve cuff or non-implanted hook electrode, which was positioned so as not to deform or raise the nerve from its bed. A Grass SD9 stimulator (Grass Astromed Technologies) was used to generate stimulation pulses for which parameters were chosen to minimize the applied voltage while maintaining a recordable and consistent EMG response; these parameters were 6 monophasic square pulses, 5 Hz, 7 V (<10 mA), 50 μs. Further, low stimulation frequency and pulse width allowed elimination of movement and tetanic effects from EMG recordings. To confirm consistency of stimulation and response before and after joint manipulation, 5 such pulse trains were executed with a 1 s gap in between each train. To avoid effects of fatigue, which could influence pulse amplitude over the 30 stimulation protocol, latencies and amplitudes were calculated from stimulation sets immediately prior to and following the leg position adjustment (Figure 4).

Recordings were taken with subdermal needle electrodes (Grass Technologies) implanted in the interosseus muscles of the foot. All recordings were amplified and filtered with a Grass CP511 AC Amplifier (Grass Technologies) set to 100× amplification and 30-3000 Hz bandpass filter. The limb was positioned for measurements as described above. Recordings were analyzed for repeatability among and between stimulation trains. Lack of repeatability could indicate surgical damage or inappropriate cuff positioning, and therefore data sets with inconsistent or non-repeatable action potentials in either joint position were discarded.

All signals were generated or recorded through National Instruments LabVIEW™, and data processing was done in MATLAB™ (MathWorks, Natick, MA).

### Statistics

Statistics were performed using MATLAB (Mathworks, Natick, MA). Data were initially analyzed for outliers using Peirce’s Criterion, a conservative and probability-based method; two points based on amplitudes and one point based on latencies were removed from the cuffed data set (final: n = 14), while none from the control required removal. Means were compared using paired t-tests or ANOVA (as indicated in subsequent text), and variances were compared using F-test. Linear regression slopes were compared using ANCOVA. Tukey’s HSD post-hoc testing was performed to compare individual groups, and accounted for multiple comparisons. Type I error (α) was set to 0.05 for all comparisons.

## Results

### Biomechanics

**Figure 5:**
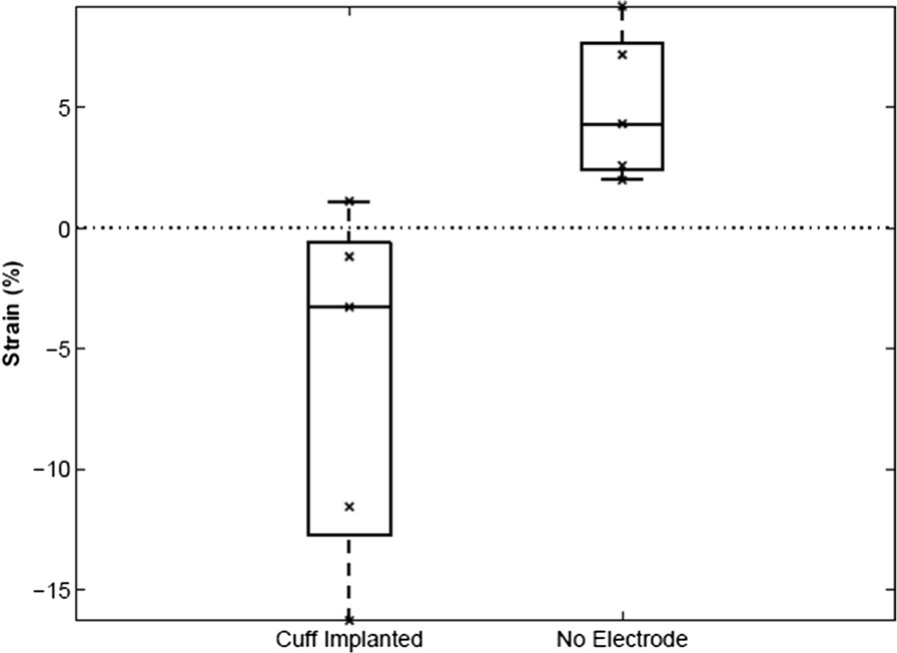
**Average strain measurements for joint rotation with the cuff implanted (left) and without (right).** Differences in the direction of stretch is obvious through the sign change. There is also a noticeable increase in range and standard deviation for the cuff versus control (no electrode).

Nerve strain was measured following knee extension, to quantify the mechanical impact of cuff implantation. Results found with and without an implanted cuff show a significant difference in strain (Figure 5, Cuff: ${\bar{x}}_{\text{cuff}}$ = -6.24%; Control: ${\bar{x}}_{\text{control}}$ = +5.08%, ANOVA p < 0.01). Interestingly, this difference is also accompanied by a sign change, suggesting that with cuff implantation, examined regions of nerves, unexpectedly, shorten with knee extension. The consistency of strain from trial to trial was also affected in cuffed nerves. F-test on the measurements with and without the implant showed no significant variance in strain with the cuff implanted (*S*_*cuff*_ = 7.39% vs. *S*_*control*_ = 3.08%; p < 0.12).

**Figure 6:**
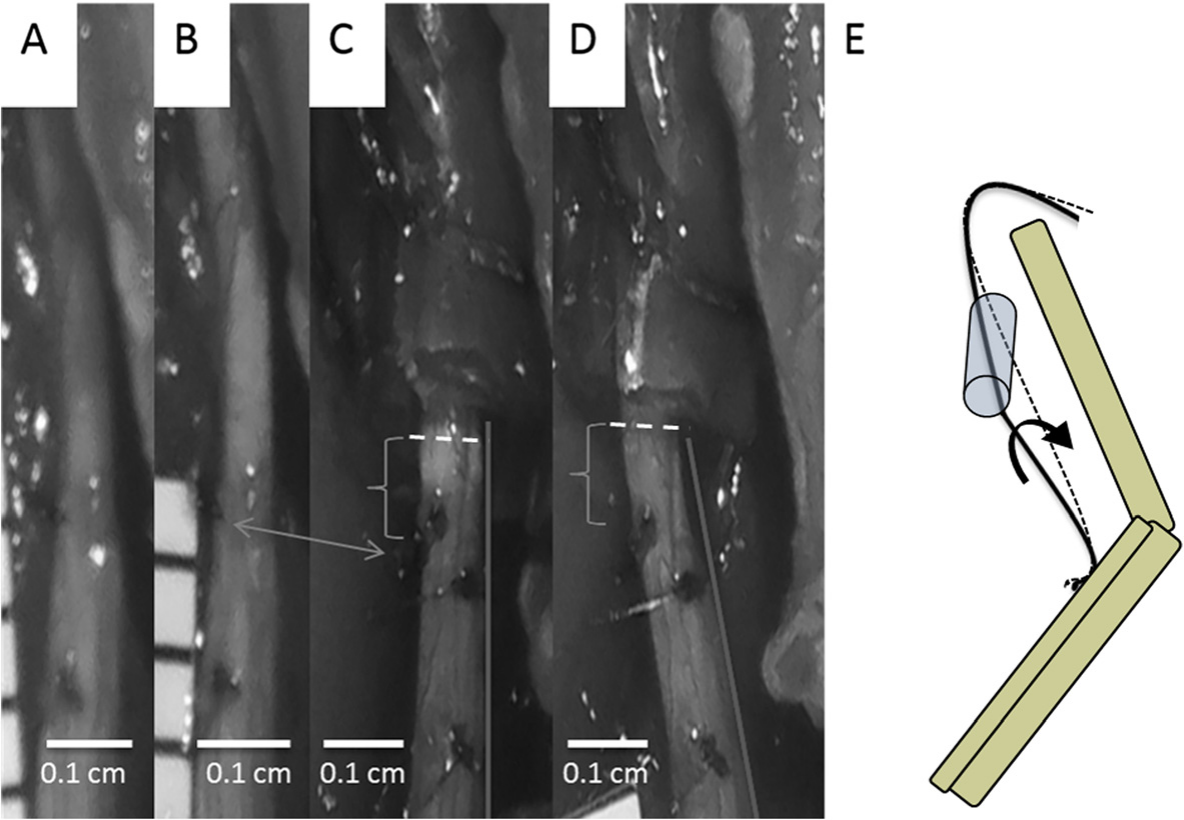
**Images taken from a single strain measurement trial and a schematic of the system.** Image definitions are as follows: **(A)** no cuff present, relaxed position; **(B)** no cuff present, extended position; **(C)** cuff present, relaxed position; **(D)** cuff present, extended position. Double headed arrow shows rotation of the nerve seen through the marker position (left side in **(B)**, left to center in **(C)**). Brackets highlight differences in the impact of nerve contact with the cuff, and dotted lines highlight the cuff-nerve interface. **(C)** shows increased compression not present in **(D)**. The line on the right of the nerve in **(C)** and **(D)** show alteration of the natural nerve path. **(E)** shows a schematic of the nerve movements seen in images **(C)** and **(D)**.

Though cuffs were carefully aligned with the nerve during implantation, as the knee joint extended, the natural motion of the nerve was dramatically restricted by the cuff (e.g., Figure 5). Thus, while we hypothesized that the cuff would induce a tethering effect that resulted in a simple lengthening of the distal nerve (Figure 1), in reality, the cuff also restricted the natural torsion of the nerve and its rotation around the hip joint during knee extension (Figure 6E). Additionally, the trajectory of the nerve as it entered and exited the nerve cuff was altered during joint movement, imposing local compression (Figure 6C & 6D). Though artifacts associated with two-dimensional imaging of a three-dimensional phenomenon precluded accurate measurement of deviation from a normal nerve trajectory, qualitative assessment suggested that this parameter was influenced by slight differences in implant location along the length of the nerve, or subtle differences in nerve geometry.

### Electrophysiology

**Figure 7:**
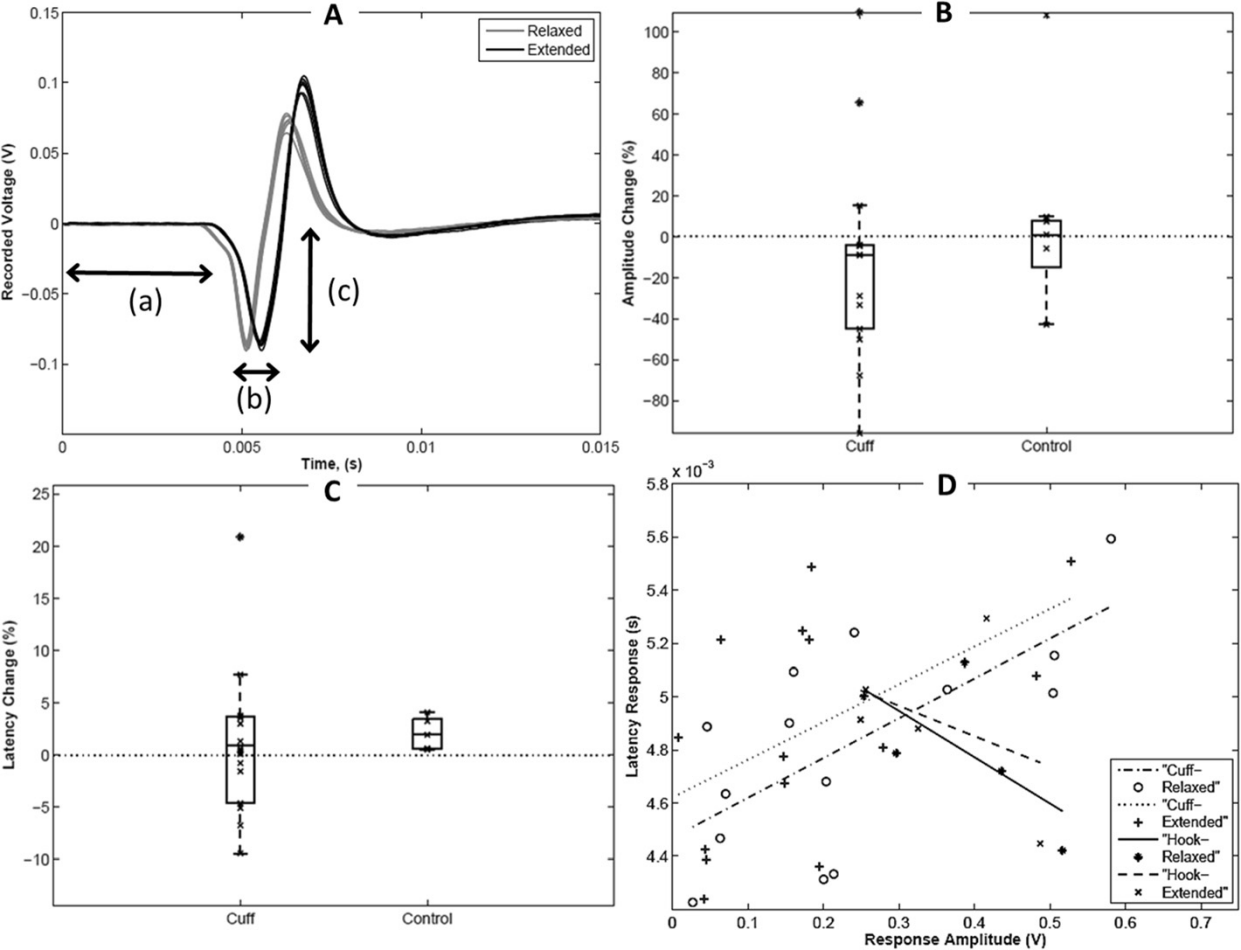
**Latency and amplitude changes from relaxed to extended in cuff versus control experiments. (A)** Superimposed Action Potentials from a Single Stimulation Set of an uncuffed control nerve. (Relaxed black and extended gray) Time t = 0 indicates the initiation of a stimulus. Double headed arrows represent measurements taken for analysis: **(a)** Latency, **(b)** Latency Difference, **(c)** Amplitude. **(B)** Box and whisker plot showing percent change in latency. Average, standard deviation, and data range differences can be seen for cuff and control experiments. **(C)** Box and whisker plot showing Amplitude difference calculations. As with latency, average, standard deviation, and data range differences can be seen for cuff and control experiments. **(D)** Regression of Latency versus amplitude showing opposing relationships for the cuff and control trials.

We next examined whether perturbations to the bio-mechanical environment induced functional alterations. Using EMG, we tested whether nerve cuff implantation caused acute differences in action potential conduction. Conduction latency and amplitudes were calculated from raw compound muscle action potential (CMAP) traces using cuff electrodes or a hook electrode control. The protocol in Figure 7A describes measurement of the amplitude as the negative peak; other protocols, such as peak-to-peak amplitude were tested and no differences were found. Knee joints were configured in two positions, corresponding to unextended and extended nerves (Figure 2B). In both positions, analyzed EMG signals maintained consistent and expected shape among and between stimulation trains, indicating repeatable measurements, an appropriate cuff-nerve interface, and reliable calculations. Such consistency was also maintained among members of the position control group, in which the position testing order was reversed.

In uncuffed control nerves (e.g. Figure 7A), a delay of 0.10 ms (2% change, Figure 7B) was observed in nerves following knee extension compared to a neutral configuration, while following cuff implantation, a delay of 0.05 ms was observed (Table 1). Two-way ANOVA revealed no effect of cuffing or knee extension on average latencies, and no interaction between these factors. Post-hoc comparisons also revealed no significant differences between experimental groups. However, a significant increase in the variance of the percent changes in latency was found in cuffed versus control nerves (F-distribution of f = 18.59, p < 0.01). The latency changes with the cuff implanted ranged in magnitude from near zero to almost twice the maximum of hook controls. Surprisingly, latencies were frequently larger in the relaxed position compared to the extended (negative values, Figure 5B). Magnitudes of amplitudes were not formally compared due to likely differences in recruitment with a cuff and hook electrode. However, a minimal average decrease in amplitude from relaxed to extended (${\bar{d}}_{1,2}$ ≈ -0.02 V) was seen for both the cuff and control trials. Again, though, amplitude changes in cuffed nerves showed a larger range and significantly higher variance than in controls (Figure 7C,F-distribution of f = 473.42, p < 1e-4).

**Table 1:** **Comparison of EMG latencies** (**in miliseconds**) **for uncuffed** (**control**) **and cuffed nerves**

**CONDITION**	**RELAXED**	**EXTENDED**
**CONTROL**	4.81 +/-0.27	4.91+/-0.31
**CUFF IMPLANTED**	4.77 +/-0.46	4.82+/-0.44

To test for coupling between differences in latency and amplitude, we performed linear regression (Figure 7D). Regression analysis produced a weak positive linear relationship between latency and amplitude in cuffed nerves, in both extended and relaxed positions (relaxed: slope = 0.0015; r^2 = 0.65; extended: slope = 0.0014; r^2 = 0.53). On the other hand, control trials showed an opposite negative relationship between latency and amplitude, in both relaxed and extended positions (relaxed: slope = -0.0017; r^2 = 0.94; extended: slope = -0.0011; r^2 = 0.93). ANCOVA analysis confirmed that cuffing, but not joint position, significantly impacted the amplitude-latency relationship (F = 6.68; p < 0.02).

## Discussion

In this work, we performed kinematic analysis and electromyography to probe acute functional influences of nerve cuff implantation. Cuff implantation constrained normal nerve movement, and resulted in a significant increase in variability in both the functional (latency and amplitude) and biomechanical (nerve strain) response; physiological movement of joints spanned by cuffed nerves may especially impact electrode performance and nerve function. We speculate that examination of such acute effects may hold the potential to ultimately predict chronic neuropathy induced by device implantation.

### Biomechanical impacts of cuff implantation

An analysis of strain following joint movement shows significantly different behaviors with and without an implant present, implying a significant shift in the mechanical environment (Figures 5, 6). Despite the absence of compression following cuff implantation in a neutral joint position, the local compression and tethering observed at the cuff edges during joint movement may seed the range of effects that has been described in chronic implantation studies, whose symptoms are analogous to entrapment neuropathies [58]. The compressive action of the cuff is likely compounded by, and in return compounds, natural inflammatory actions due to the presence of a foreign body. Reported responses to compressive effects include axonal degeneration, demyelination, fibrosis, and are sometimes associated with a loss in electrophysiological function [2], [35-45].

It should be noted that despite the possible repercussions in altering the natural biomechanical environment in the short term, several long-term studies show at least some recovery [37], [42], [43] with remyelination, axonal regeneration, decreased fibrosis [37], and recovery of function [36], [42]. Tissue encapsulation of the cuff, a common effect [2], [35-45], may serve to stabilize the cuff over the life of the implant and lead to growth and partial recovery of damage done to the nerve [43]. Nerve growth has been shown to be positively influenced by strains induced under natural conditions [59], [60]; as a result, it is possible that a sustained increase in nerve tension during chronic cuff implantation may promote mechanically induced growth pathways within the axon leading to ameliorative growth [61].

### Electrophysiological impacts of biomechanical changes

Electrophysiological testing showed consistent, stable and expected CMAP shape, but revealed significant differences in action potential conduction with implantation of cuff electrodes compared with controls. Specifically, latency and amplitude measurements showed a significant increase in variability with the cuff implanted. The regression of EMG latency and amplitude given in Figure 7D provides an interesting contrast between cuffed and uncuffed nerves, and some insight into the nature of the interaction between the cuff and the nerve. The negative relationship in control trials is consistent with the expected decrease in latency with increased amplitude. This relationship is likely due to increased conduction velocities in larger diameter fibers, which innervate larger muscle motor units [62]. The positive relationship found between latency and amplitude following cuff implantation is more complex. We speculate that minimal changes in latency, but higher reductions in amplitude (i.e., left side of regression plot in Figure 7D) are consistent with clinical changes following acute nerve compression over a considerable length of the nerve [63]. This may reflect suppression of conduction in more superficial axons, but not in less compressed axons deeper within the nerve. On the opposite end of the correlation (i.e., right side of regression plot in Figure 7B), highly localized impingement could explain increased latency, resulting from local inefficiency in transmission due to focal ischemia or selective loss of large fibers, but maintenance of compound conduction, and thus amplitude, proximal and distal to the site of tethering [64], [65].

### Implications for device evaluation and design

On one hand, within reason, it is possible to safely exploit the flexibility of the nerve to increase device performance. Recently developed, an elliptically shaped cuff electrode reshapes the circular nerve by flattening it into a more elliptical shape, effectively reducing the minor axis of the nerve and allowing for isolation of specific fiber bundles which have been pushed towards the electrode surface [33], [66]. On the other hand, our findings indicate that the biomechanical environment of the nerve-device interface may adversely impact device performance and nerve function. We propose that the high variance present in our data is likely due to variations in the mechanical impact of the cuff on the nerve, which is reliant on the relationship with the cuff and the nerve as well as the cuff and the surrounding musculature. Consistency in implant location is a critical aspect of implantations, but adaptations may also be necessary to match variability in nerve and musculature between patients. Studies of clinical implantations of vagal nerve stimulators have shown significant mechanical impact including tensile and compressive loading [67-70] as well as functional changes such as vocal function, voice alteration, dyspnea, coughing and aspiration [24], [25], [46]. Some severity in the resulting functional alterations has been alleviated by better matching lead coil diameter to nerve diameter [71].

Future cuff designs may also reduce mechanical perturbations through utilization of materials more closely suited to the mechanical properties of the nerve tissue. The epidermal microelectronics, developed by Kim et al. [72], are designed to match the mechanical properties of human skin to allow long term adhesion and comfort to subjects. Similarly, collagen matrix cuffs have been designed as conduits for growth and repair of damaged nerve tissue. These cuffs match the natural mechanical properties of peripheral nerves, but are also designed to slowly incorporate themselves into the epineurium and surrounding tissues. Ideally, after long term implantation, the effect of the cuff would be negligible and only the mechanical presence of the leads would remain, leaving behind a markedly reduced device footprint [73], [74]. Progress is still required in development of devices able to stimulate without long lead wires, which have been shown to migrate and tug on nerves in clinical applications [24], [25], [43], [46]. This goal may be realized as wireless power transfer technologies become more widely applicable to biomedical applications.

## Conclusion

The altered mechanical environment created by the presence of a cuff electrode has significant acute effects on nerve functionality; future studies will directly correlate acute function with chronic neural structure and function. This study provides an important first step in formally examining the reciprocal relationships between nerve mechanics, nerve architecture, and device implantation, towards more effective peripheral nerve device design and evaluation.

## Competing interests

The authors declare that they have no competing interests.

## Author Contributions

SR, VK, and SBS contributed to the conception and design of research; SR, EA, and KW performed experiments; SR, EA, and SBS analyzed data and interpreted results of experiments; SR prepared figures and drafted manuscript; All authors edited and approved final version of manuscript.

## References

[B1] Rodriguez FJ Ceballos D Schüttler M Valero A Valderrama E Stieglitz T Navarro X Polyimide cuff electrodes for peripheral nerve stimulation J Neurosci Methods 2000 98 105 118 10880824 10.1016/s0165-0270(00)00192-8

[B2] Loeb GE Peck RA Cuff electrodes for chronic stimulation and recording of peripheral nerve activity J Neurosci Methods 1996 64 95 103 8869489 10.1016/0165-0270(95)00123-9

[B3] Garde K Keefer E Botterman B Galvan P Romero MI Early Interfaced Neural Activity from Chronic Amputated Nerves Front Neuroengineering 2009 2 5 10.3389/neuro.16.005.2009 PMC2691654 19506704

[B4] Fugleholm K Schmalbruch H Krarup C Early peripheral nerve regeneration after crushing, sectioning, and freeze studied by implanted electrodes in the cat J Neurosci 1994 14 2659 2673 8182434 10.1523/JNEUROSCI.14-05-02659.1994 PMC6577445

[B5] Lawrence SM Dhillon GS Horch KW Fabrication and characteristics of an implantable, polymer-based, intrafascicular electrode J Neurosci Methods 2003 131 9 26 14659819 10.1016/s0165-0270(03)00231-0

[B6] Lawrence SM Dhillon GS Jensen W Yoshida K Horch KW Acute peripheral nerve recording Characteristics of polymer-based longitudinal intrafascicular electrodes IEEE Trans Neural Syst Rehabil Eng 2004 12 345 348 15473197 10.1109/TNSRE.2004.831491

[B7] Micera S Navarro X Carpaneto J Citi L Tonet O Rossini PM Carrozza MC Hoffmann KP Vivo M Yoshida K Dario P On the Use of Longitudinal Intrafascicular Peripheral Interfaces for the Control of Cybernetic Hand Prostheses in Amputees IEEE Trans Neural Syst Rehabil Eng 2008 16 453 472 18990649 10.1109/TNSRE.2008.2006207

[B8] Navarro X Lago N Vivo M Yoshida K Koch KP Poppendieck W Micera S Neurobiological evaluation of thin-film longitudinal intrafascicular electrodes as a peripheral nerve interface Rehabi Robot, ICORR 2007 IEEE 10th Int Conf 2007 2007 643 649

[B9] Badia J Boretius T Pascual-Font A Udina E Stieglitz T Navarro X Biocompatibility of Chronically Implanted Transverse Intrafascicular Multichannel Electrode (TIME) in the Rat Sciatic Nerve IEEE Trans Biomed Eng 2011 58 2324 2332 10.1109/TBME.2011.2153850 21571604

[B10] Boretius T Badia J Pascual-Font A Schuettler M Navarro X Yoshida K Stieglitz T A transverse intrafascicular multichannel electrode (TIME) to interface with the peripheral nerve Biosens Bioelectron 2010 26 62 69 20627510 10.1016/j.bios.2010.05.010

[B11] Branner A Normann RA A multielectrode array for intrafascicular recording and stimulation in sciatic nerve of cats Brain Research Bulliten 2000 51 293 306 10.1016/s0361-9230(99)00231-2 10704779

[B12] Branner A Stein RB Normann RA Selective stimulation and recording using a slanted multielectrode array Eng Med Biol 1999 21st Annual Conf the 1999 Annual Fall Meeting of the Biomed Eng Soc] BMES/EMBS Conf 1999 371 377

[B13] Branner A Stein RB Normann RA Selective Stimulation of Cat Sciatic Nerve Using an Array of Varying-Length Microelectrodes J Neurophysiol 2001 85 1585 1594 11287482 10.1152/jn.2001.85.4.1585

[B14] Clark GA Ledbetter NM Warren DJ Harrison RR Recording sensory and motor information from peripheral nerves with Utah Slanted Electrode Arrays Eng Med Biol Soc EMBC, 2011 Annual Int Conf the IEEE 2011 2011 4641 4644 10.1109/IEMBS.2011.6091149 22255372

[B15] Delbeke J Oozeer M Veraart C Position, size and luminosity of phosphenes generated by direct optic nerve stimulation Vision Res 2003 43 1091 1102 12676250 10.1016/s0042-6989(03)00013-0

[B16] Delbeke J Wanet-Defalque MC Gérard B Troosters M Michaux G Veraart C The microsystems based visual prosthesis for optic nerve stimulation Artif Organs 2012 26 232 234 11940020 10.1046/j.1525-1594.2002.06939.x

[B17] Veraart C Raftopoulos C Mortimer JT Delbeke J Pins D Michaux G Vanlierde A Parrini S Wanet-Defalque M-C Visual sensations produced by optic nerve stimulation using an implanted self-sizing spiral cuff electrode Brain Res 1998 813 181 186 9824694 10.1016/s0006-8993(98)00977-9

[B18] Veraart C Wanet-Defalque MC Gérard B Vanlierde A Delbeke J Pattern Recognition with the Optic Nerve Visual Prosthesis Artif Organs 2012 27 996 1004 14616518 10.1046/j.1525-1594.2003.07305.x

[B19] DeGiorgio CM Schachter SC Handforth A Salinsky M Thompson J Uthman B Reed R Collins S Tecoma E Morris GL Vaughn B Naritoku DK Henry T Labar D Gilmartin R Labiner D Osorio I Ristanovic R Jones J Murphy J Ney G Wheless J Lewis P Heck C Prospective Long‒Term Study of Vagus Nerve Stimulation for the Treatment of Refractory Seizures Epilepsia 2012 41 1195 1200 10999559 10.1111/j.1528-1157.2000.tb00325.x

[B20] Krahl SE Clark KB Smith DC Browning RA Locus Coeruleus Lesions Suppress the Seizure‒Attenuating Effects of Vagus Nerve Stimulation Epilepsia 2012 39 709 714 9670898 10.1111/j.1528-1157.1998.tb01155.x

[B21] Krahl SE Senanayake SS Pekary AE Sattin A Vagus nerve stimulation (VNS) is effective in a rat model of antidepressant action J Psychiatr Res 2004 38 237 240 15003428 10.1016/j.jpsychires.2003.11.005

[B22] Naritoku DK Terry WJ Helfert RH Regional induction of fos immunoreactivity in the brain by anticonvulsant stimulation of the vagus nerve Epilepsy Res 1995 22 53 62 8565967 10.1016/0920-1211(95)00035-9

[B23] Takaya M Terry WJ Naritoku DK Vagus Nerve Stimulation Induces a Sustained Anticonvulsant Effect Epilepsia 2012 37 111 1116 10.1111/j.1528-1157.1996.tb01033.x 8917063

[B24] Lundgren J Amark P Blennow G Stromblad LG Wallstedt L Vagus nerve stimulation in 16 children with refractory epilepsy Epilepsia 1998 39 809 813 9701369 10.1111/j.1528-1157.1998.tb01173.x

[B25] Lundy DS Casiano RR Landy HJ Gallo J Gallo B Ramsey RE Effects of vagal nerve stimulation on laryngeal function J Voice 1993 7 359 364 8293068 10.1016/s0892-1997(05)80259-0

[B26] Sahin M Durand DM Improved nerve cuff electrode recordings with subthreshold anodic currents IEEE Trans Biomed Eng 1998 45 1044 1050 9691579 10.1109/10.704873

[B27] Sahin M Haxhiu MA Durand DM Dreshaj IA Sahin M Haxhiu MA Durand DM Dreshaj IA Spiral nerve cuff electrode for recordings of respiratory output J Appl Physiol 1997 83 317 322 9216978 10.1152/jappl.1997.83.1.317

[B28] DiMarco AF Onders RP Kowalski KE Miller ME Ferek S Mortimer JT Phrenic Nerve Pacing in a Tetraplegic Patient via Intramuscular Diaphragm Electrodes Am J Respir Crit Care Med 2002 166 1604 1606 12471076 10.1164/rccm.200203-175CR

[B29] Weese-Mayer DE Silvestri JM Kenny AS Ilbawi MN Hauptman SA Lipton JW Talonen PP Garcia HG Watt JW Exner G Baer GA Elefteriades JA Peruzzi WT Alex CG Harlid R Vincken W Davis GM Decramer M Kuenzle C Saeterhaug A Schöber JG Diaphragm Pacing with a Quadripolar Phrenic Nerve Electrode: An International Study Pacing Clin Electrophysiology 2012 19 1311 1319 10.1111/j.1540-8159.1996.tb04209.x 8880794

[B30] Gustafson KJ Pinault GCJ Neville JJ Syed I Davis JA Jean-Claude J Triolo RJ Fascicular anatomy of human femoral nerve: Implications for neural prostheses using nerve cuff electrodes J Rehabil Res Dev 2009 46 973 984 20104420 10.1682/jrrd.2008.08.0097 PMC2967182

[B31] Navarro X Krueger TB Lago N Micera S Stieglitz T Dario P A critical review of interfaces with the peripheral nervous system for the control of neuroprostheses and hybrid bionic systems J Peripher Nerv Syst 2012 10 229 258 16221284 10.1111/j.1085-9489.2005.10303.x

[B32] Frankel MA Dowden BR Mathews VJ Normann RA Clark GA Meek SG Multiple-Input Single-Output Closed-Loop Isometric Force Control Using Asynchronous Intrafascicular Multi-Electrode Stimulation IEEE Trans Neural Syst Rehabil Eng 2011 19 325 332 21385670 10.1109/TNSRE.2011.2123920

[B33] Caparso AV Durand DM Mansour JM Caparso AV Durand DM Mansour JM A Nerve Cuff Electrode for Controlled Reshaping of Nerve Geometry J Biomater Appl 2009 24 247 273 18987020 10.1177/0885328208097426 PMC3569731

[B34] Polasek KH Hoyen HA Keith MW Tyler DJ Human Nerve Stimulation Thresholds and Selectivity Using a Multi-contact Nerve Cuff Electrode IEEE Trans Neural Syst Rehabil 2007 15 76 82 Eng 10.1109/TNSRE.2007.891383 17436879

[B35] Edell DJ Churchill JN Gourley IM Biocompatibility of a Silicon Based Peripheral Nerve Electrode Biomater Med Devices Artif Organs 1982 10 103 122 7139019 10.3109/10731198209118775

[B36] Murphy B Krieger C Hoffer J-A Chronically implanted epineural electrodes for repeated assessment of nerve conduction velocity and compound action potential amplitude in rodents J Neurosci Methods 2004 132 25 33 14687672 10.1016/j.jneumeth.2003.08.013

[B37] Larsen JO Thomsen M Haugland M Sinkjaer T Degeneration and Regeneration in rabbit peripheral nerve with long-term nerve cuff electrode implant: a stereological study of myelinated and unmyelinated axons Acta Neuropathol 1998 96 365 378 9797001 10.1007/s004010050907

[B38] Grill WM Mortimer JT Electrical Properties of Implant Encapsulation Tissue Ann Biomed Eng 1994 22 23 33 8060024 10.1007/BF02368219

[B39] Leventhal DK Cohen M Durand DM Chronic histological effects of the flat interface nerve electrode J Neural Eng 2006 3 102 16705266 10.1088/1741-2560/3/2/004

[B40] Vince V Brelen ME Delbeke J Colin IM Anti-TNF-α reduces the inflammatory reaction associated with cuff electrode implantation around the sciatic nerve J Neuroimmunol 2005 165 121 128 15951027 10.1016/j.jneuroim.2005.04.019

[B41] Vince V Thil M-A Gérard A-C Veraart C Delbeke J Colin IM Cuff electrode implantation around the sciaticnerve is associated with an upregulation of TNF-α and TGF-β1 J Neuroimmunol 2005 159 75 86 15652405 10.1016/j.jneuroim.2004.10.010

[B42] Thil M-A Duy DT Colin IM Delbeke J Timecourse of tissue remodelling and electrophysiology in the rat sciatic nerve after spiral cuff electrode implantation J Neuroimmunol 2007 185 103 114 17343923 10.1016/j.jneuroim.2007.01.021

[B43] Grill WM Mortimer JT Neural and connective tissue response to long-term implantation of multiple contact nerve cuff electrodes J Biomed Mater Res 2000 50 215 226 10679687 10.1002/(sici)1097-4636(200005)50:2<215::aid-jbm17>3.0.co;2-a

[B44] Gupta R Nassiri N Hazel A Bathen M Mozaffar T Chronic nerve compression alters Schwann cell myelin architecture in a murine model Muscle Nerve 2012 45 231 241 22246880 10.1002/mus.22276 PMC3262776

[B45] Gupta R Rowshan K Chao T Mozaffar T Steward O Chronic nerve compression induces local demyelination and remyelination in a rat model of carpal tunnel syndrome Exp Neurol 2004 187 500 508 15144876 10.1016/j.expneurol.2004.02.009

[B46] Zumsteg D Jenny D Wieser HG Vocal cord adduction during vagus nerve stimulation for treatment of epilepsy Neurology 2000 54 1388 1389 10746619 10.1212/wnl.54.6.1388

[B47] Boyd BS Puttlitz C Gan J Topp KS Strain and excursion in the rat sciatic nerve during a modified straight leg raise are altered after traumatic nerve injury J Orthop Res 2012 23 764 770 16022988 10.1016/j.orthres.2004.11.008

[B48] Topp KS Boyd BS Structure and Biomechanics of Peripheral Nerves: Nerve Responses to Physical Stresses and Implications for Physical Therapist Practice Phys Ther 2006 86 92 109 16386065 10.1093/ptj/86.1.92

[B49] Wright TW Glowczewskie F Jr Cowin D Wheeler DL Ulnar nerve excursion and strain at the elbow and wrist associated with upper extremity motion J Hand Surg [Am] 2001 26 655 662 10.1053/jhsu.2001.26140 11466640

[B50] Wright TW Glowczewskie F Wheeler D Miller G Cowin D Excursion and strain of the median nerve J Bone Joint Surg Am 1996 78 1897 1903 8986667 10.2106/00004623-199612000-00013

[B51] Wall EJ Kwan MK Rydevik BL Woo SL Garfin SR Stress relaxation of a peripheral nerve J Hand Surg [Am] 1991 16 859 863 10.1016/s0363-5023(10)80149-2 1940164

[B52] Kwan MK Wall EJ Massie J Garfin SR Strain, stress and stretch of peripheral nerve. Rabbit experiments in vitro and in vivo Acta Orthop Scand 1992 63 267 272 1609588 10.3109/17453679209154780

[B53] Phillips JB Smit X De Zoysa N Afoke A Brown RA Peripheral nerves in the rat exhibit localized heterogeneity of tensile properties during limb movement J Physiol 2004 557 879 887 15064329 10.1113/jphysiol.2004.061804 PMC1665165

[B54] Wall E Massie J Kwan M Rydevik B Myers R Garfin S Wall E Massie J Kwan M Rydevik B Myers R Garfin S Experimental stretch neuropathy. Changes in nerve conduction under tension J Bone Joint Surg (Br) 1992 74 126 129 1732240 10.1302/0301-620X.74B1.1732240

[B55] Highet WB Sanders FK The effects of stretching nerves after suture Br J Surg 2012 30 355 369

[B56] Naples GG Mortimer JT Scheiner A Sweeney JD A spiral nerve cuff electrode for peripheral nerve stimulation IEEE Trans on Biomed Eng 1988 35 905 916 10.1109/10.8670 3198136

[B57] Rydevik BL Kwan MK Myers RR Brown RA Triggs KJ Woo SL Garfin SR An in vitro mechanical and histological study of acute stretching on rabbit tibial nerve J Orthop Res 1990 8 694 701 2388109 10.1002/jor.1100080511

[B58] Rempel DM Diao E Entrapment neuropathies: pathophysiology and pathogenesis J Electromyogr Kinesiol 2004 14 71 75 14759752 10.1016/j.jelekin.2003.09.009

[B59] Nishiura Y Hara Y Yoshii Y Ochiai N Gradual stretching of the proximal nerve stump induces the growth of regenerating sprouts in rats J Orthop Res 2008 26 1012 1017 18327803 10.1002/jor.20587

[B60] Nishiura Y Yamada Y Hara Y Ichimura H Yoshii Y Ochiai N Repair of peripheral nerve defect with direct gradual lengthening of the proximal nerve stump in rats J Orthop Res 2006 24 2246 2253 17013872 10.1002/jor.20280

[B61] Loverde JR Ozoka VC Aquino R Lin L Pfister BJ Live imaging of axon stretch growth in embryonic and adult neurons J Neurotrauma 2011 28 2389 2403 21663384 10.1089/neu.2010.1598

[B62] Kupa EJ Roy SH Kandarian SC Luca CJD Effects of muscle fiber type and size on EMG median frequency and conduction velocity J Appl Physiol 1995 79 23 32 7559225 10.1152/jappl.1995.79.1.23

[B63] Stecker MM Baylor K Chan YM Acute nerve compression and the compound muscle action potential J Brachial Plex and Peripher Nerve Inj 2008 3 1 18211681 10.1186/1749-7221-3-1 PMC2245939

[B64] Parry GJ Cornblath DR Brown MJ Transient conduction block following acute peripheral nerve ischemia Muscle Nerve 1985 8 409 412 16758587 10.1002/mus.880080510

[B65] Kiechl S 2013. Clinical and Electrodiagnostic Work-up of Peripheral Nerve Lesions Medical Radiology, Diagnostic Imaging: High-Resolution Sonography of the Peripheral Nervous System Berlin 2008 Heidelberg: Springer-Verlag Berlin Heidelberg 43 70

[B66] Schiefer MA Polasek KH Triolo RJ Pinault GCJ Tyler DJ Selective stimulation of the human femoral nerve with a flat interface nerve electrode J Neural Eng 2010 7 26006 20208125 10.1088/1741-2560/7/2/026006 PMC2915830

[B67] Pearl PL Conry JA Yaun A Taylor JL Heffron AM Sigman M Tsuchida TN Elling NJ Bruce DA Gaillard WD Misidentification of vagus nerve stimulator for intravenous access and other major adverse events Pediatr Neurol 2008 38 248 251 18358402 10.1016/j.pediatrneurol.2007.12.002

[B68] Ng WH Donner E Go C Abou-Hamden A Rutka JT Revision of vagal nerve stimulation (VNS) electrodes: review and report on use of ultra-sharp monopolar tip Childs Nerv Syst 2010 26 1081 1084 20225085 10.1007/s00381-010-1121-2

[B69] O’Neill BR Wilberger JE Revision of vagal nerve stimulator electrodes through a posterior cervical triangle approach: technical note Neurosurgery 2010 67 457 460 21099572 10.1227/NEU.0b013e3181f825a3

[B70] Ortler M Unterhofer C Dobesberger J Haberlandt E Trinka E Complete removal of vagus nerve stimulator generator and electrodes J Neurosurg Pediatr 2010 5 191 194 20121370 10.3171/2009.9.PEDS0810

[B71] Santos PM Evaluation of laryngeal function after implantation of the vagus nerve stimulation device Otolaryngol Head Neck Surg 2003 129 269 273 12958579 10.1016/S0194-5998(03)00605-3

[B72] Kim DH Lu N Ma R Kim YS Kim RH Wang S Wu J Won SM Tao H Islam A Yu KJ Kim TI Chowdhury R Ying M Xu L Li M Chung HJ Keum H McCormick M Liu P Zhang YW Omenetto FG Huang Y Coleman T Rogers JA Epidermal Electronics Science 2011 333 838 843 21836009 10.1126/science.1206157

[B73] Farole A Jamal BT A Bioabsorbable Collagen Nerve Cuff (NeuraGen) for Repair of Lingual and Inferior Alveolar Nerve Injuries: A Case Series J Oral Maxillofac Surg 2008 66 2058 2062 18848102 10.1016/j.joms.2008.06.017

[B74] Archibald SJ Krarup C Shefner J Li ST Madison RD A collagen‒based nerve guide conduit for peripheral nerve repair: An electrophysiological study of nerve regeneration in rodents and nonhuman primates J Comp Neurol 2012 306 685 696 2071700 10.1002/cne.903060410

